# Overseas GP recruitment: comparing international GP training with the UK and ensuring that registration standards and patient safety are maintained

**DOI:** 10.3399/bjgpopen18X101640

**Published:** 2019-04-17

**Authors:** Emily Fletcher, Anna Sansom, Emma Pitchforth, Gerens Curnow, Adrian Freeman, Kamila Hawthorne, John Campbell

**Affiliations:** 1 Research Fellow, College Of Medicine And Health, University of Exeter Medical School, St Luke’s Campus, Exeter, UK; 2 Research Fellow, College Of Medicine And Health, University of Exeter Medical School, St Luke’s Campus, Exeter, UK; 3 Senior Lecturer and Senior Research Fellow, College Of Medicine And Health, University of Exeter Medical School, St Luke’s Campus, Exeter, UK; 4 Medical Student, University of Exeter Medical School, St Luke’s Campus, Exeter, UK; 5 Professor of Medical Education, College Of Medicine And Health, University of Exeter Medical School, St Luke’s Campus, Exeter, UK; 6 Vice Chair (Professional Development), Royal College of General Practitioners, London, UK; 7 Professor of General Practice and Primary Care, College Of Medicine And Health, University of Exeter Medical School, St Luke’s Campus>, Exeter, UK

**Keywords:** Postgraduate education, Licensing, appraisal & revalidation, Research methods (other), General practice, Primary heath care, Workforce

## Abstract

**Background:**

Ambitious overseas recruitment targets have been set by the UK government to help alleviate the current GP shortage. European Economic Area (EEA) doctors can join the UK’s GP register under European law. Non-EEA doctors must obtain a Certificate of Eligibility for General Practice Registration (CEGPR), demonstrating equivalence to UK-trained doctors. CEGPR applications can be time-consuming and burdensome. To meet overseas recruitment targets, it is important to facilitate the most efficient route into UK general practice while maintaining registration standards and patient safety.

**Aim:**

To develop a methodology to map postgraduate GP training and healthcare contextual data from an overseas country to the UK.

**Design & setting:**

Desk-based research and stakeholder interviews.

**Method:**

Four stages were undertaken: 1) developing a data collection template; 2) conducting a case study (using Australia as a test case); 3) refining the data collection template; and 4) creating a mapping framework. The case study used the 2016 curricula for the UK and Australia.

**Results:**

Five ‘domains’ were identified: healthcare context, training pathway, curriculum, assessment, and continuing professional development (CPD) and revalidation. The final data collection template comprised 49 mapping items across the domains. The methodology incorporated the application of a red, amber, or green (RAG) rating to indicate similarity of data across the five domains. Australia was rated ‘green’ for training pathway, curriculum, and assessment, and ‘amber’ for healthcare context and CPD and revalidation. The overall rating was ‘green’.

**Conclusion:**

Implementing this systematic methodology for mapping GP training between countries may support the UK’s ambitions to recruit more GPs, and alleviate current GP workforce pressures.

## How this fits in

In the context of GP workforce challenges and efforts to recruit doctors from overseas, this is the first study specifically focusing on developing a methodology for the comparison of international postgraduate GP training and healthcare context with that of the UK. The test case of Australia was used in order to develop a systematic approach that would stand up to academic scrutiny. The research provides information to guide future decisions, and direction about determining the most efficient and least burdensome route for overseas doctors to enter UK general practice while maintaining patient safety and registration standards.

## Introduction

The UK is currently experiencing a shortage of GPs, as are many countries worldwide.^1^ Since 2017 there has been a revised target from NHS England to recruit an additional 5000 GPs, with 2000 of these GPs to come from overseas.^2^ Further, it has recently been agreed that, in fact, 6000 GPs are now needed.^3,4^ These targets come in the context of falling numbers of GPs, failure to fill training posts currently available, and wider workforce challenges in the UK.^5,6^

Overseas recruitment of GPs entails a process for international doctors to apply to join the GP register of the UK’s General Medical Council (GMC), the organisation which sets the standards for delivery of medical education and training.^7^ The training of EEA doctors is recognised in the UK under European law, and these doctors are given automatic recognition which allows them to join the UK GP register. However, doctors from non-EEA countries are currently required to obtain a CEGPR in order to join the GP register.^8^

The CEGPR application process^8^ requires candidates to show that their knowledge, skills, and experience are equivalent to those of doctors who have completed an approved training programme and who have obtained a Certificate of Completion of Training (CCT) in the UK. The application involves an initial assessment by the GMC, then the doctor providing documented evidence of training, qualifications, and experience, assessed by the Royal College of General Practitioners (RCGP), which returns it to the GMC with a recommendation, with a final decision made by the GMC. Documented evidence must be authenticated or validated, and anonymised. Non-English documents must first be translated. Guidance suggests that applications contain around 500–800 pages of evidence,^9^ but most exceed this.

The combination of the burden of current CEGPR requirements and ambitious targets for overseas GP recruitment prompted NHS England (working with the RCGP and the GMC) to review assessment processes for GPs trained outside of the EEA. Their aim was to identify whether the GP registration process could be streamlined for doctors from non-EEA countries with training and experience that are seen as largely similar to the UK GP programme. A streamlined CEGPR process for non-EEA GPs would aim to make the application process quicker and less burdensome for applicants and assessors, without compromising patient safety and GP registration standards, and to enable timely recruitment of overseas GPs to help ease the overall system-wide pressures currently experienced in UK general practice.

Although published work exists relating to comparison of primary school education curricula in England to those of other countries,^10^ to the authors’ knowledge, no prior work has explored the potential for mapping GP training between different countries and the UK. The Primary Care Research Group, within the University of Exeter Medical School, worked collaboratively with the RCGP to develop and pilot a methodology to map GP training and other relevant healthcare system-contextual data from another country to the UK. The aim of the research was to provide information to guide future decisions, and direction about determining the most efficient and least burdensome route for overseas doctors to enter UK general practice, while maintaining patient safety and registration standards.

This article reports the development of a screening tool to determine a) whether there was sufficient evidence of equivalence for a country to be considered for a streamlined CEGPR process; and b) if so, to identify the specific areas of similarity and difference, to help inform the content of the streamlined process for that country.

## Method

The methods involved a rapid review of existing policies, procedures, and publications that were relevant for designing a practical approach to assessing the comparability of GP education and training of another country with the UK. A test case (Australia) was used to refine the methodology and to assess its plausibility.

**Figure 1:**
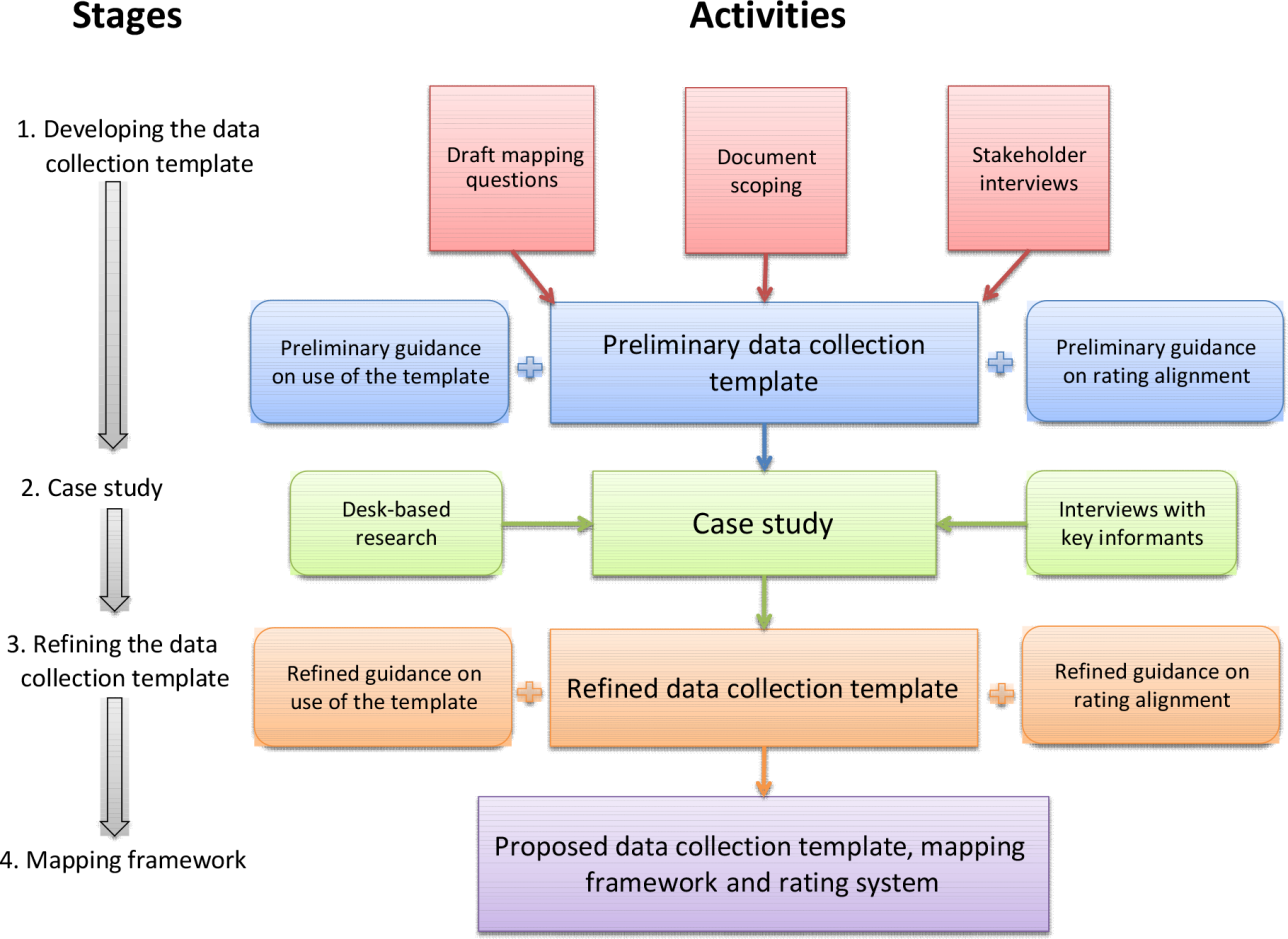
Stages and activities involved in developing and piloting the mapping methodology

A four-stage approach was taken to develop, pilot, and refine the mapping methodology (Figure 1). The work was undertaken over a period of 6 months: 4 months for development of the methods and conducting the mapping, and a further 2 months for reporting.

### Stage 1: Developing the data collection template

Three activities were conducted to identify what elements of general practice needed to be included in the mapping:

1. A list of draft mapping questions and topics was provided by the RCGP which included: GP training pathway, assessment during GP training, clinical competence and experience with population groups during and after training, CPD requirements, and revalidation systems.
2. Key documents and web-based resources were identified through internet-based searches to help ensure any further key areas of interest were identified. Published academic and grey literature was searched using the bibliographic database PubMed and Google Scholar search engine. Internet searching was used to identify governmental and non-governmental organisations and agencies with a remit in the area of primary care, GP training, assessment, and revalidation in the countries under review.
3. Stakeholder interviews were conducted to further inform the development of the preliminary template. Stakeholders were identified by the research team and invited, by email, to interview. Telephone interviews were conducted with six stakeholders, purposively sampled based on their role within the RCGP, the GMC, Health Education England (HEE), or a deanery. A semi-structured interview schedule was developed following review of the draft mapping questions document and identification of key areas of interest from the initial document-scoping exercise. Additional participant-specific questions were added to this generic schedule. Broadly, interviewees were asked what they would expect to see included in the mapping questions to feel confident in the final mapping framework. Interviews were audio-recorded (with permission) to aid notetaking and transcription. Formal qualitative analysis was not conducted due to the small sample and the time-limited nature of the work. Key topics were extracted from the interview notes and transcripts, using the categories and questions from the interview schedule and the additional issues identified by the interviewees.

**Figure 2:**
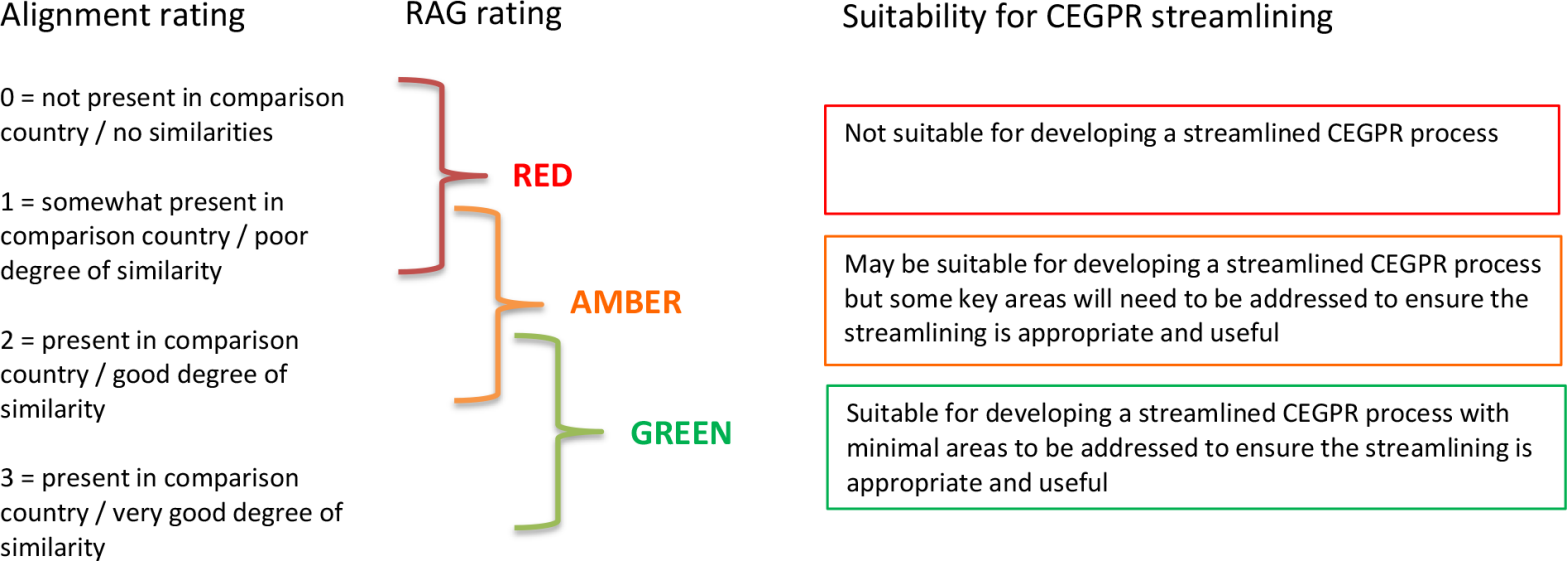
Guidance for applying an alignment rating, RAG rating, and what these mean regarding suitability for CEGPR streamlining CEGPR = Certificate of Eligibility for General Practice Registration. RAG = red, amber, green.

A preliminary data collection template was developed using the elements identified in the steps above. Guidance on the use of this template was developed, as well as a rating system for reviewing the alignment between the UK and the other country for each mapping topic. The alignment rating system used a four-point scoring system for each question, and a RAG rating for each domain and for the country overall (Figure 2).

### Stage 2: Case study

A case study was conducted to pilot the data collection template, guidance, and rating system. It was agreed by stakeholders that the pilot be completed using Australia as the country to be compared with the UK. Australia was selected for reasons including: the spoken language is English, training systems and approach to family practice were anticipated to be broadly similar to the UK, and the RCGP had some evidence that some Australian GPs would be interested in working in the UK.

The case study involved desk-based research and interviews with key informants. Desk-based research was conducted by four members of the study team. Researchers were asked to a) answer the mapping question for the UK, b) answer the question for the comparison country, c) provide a commentary on the key differences and similarities between the two countries, and d) assign alignment scores and RAG ratings. Use of tables and diagrams aided this process for many of the questions, enabling direct visual comparisons to be made. An audit trail of data sources was kept, to record how easy or difficult it was to collect the data for each mapping question, and to record how confident the researcher was that the question had been fully answered. These details were used to help refine the data collection template in stage 3.

Key informant interviews were conducted by two members of the study team, to clarify understanding and to address questions that could not be answered through desk-based research. Key informants were identified for each country using the research team’s professional networks and through official websites. Interviewees were selected purposively and invited by email. The interview schedule was structured to address the specific outstanding mapping questions and answers, and the key informant’s area of expertise. Interviews were conducted by telephone or Skype, and were audio-recorded (with permission) to aid notetaking.

### Stage 3: Refining the data collection template

The mapping questions within the data collection template were refined and reworded in an iterative process during the course of, and following completion of, the case study. The researchers discussed their experiences of completing the case study at team meetings, and used these discussions to agree the refined template and associated guidance and ratings.

### Stage 4: Creating the mapping framework

The mapping domains and questions from the refined template were used to produce the overlaying mapping framework. Two alternative frameworks were also offered for consideration.

## Results

### Stage 1: Developing the data collection template

**Figure 3:**
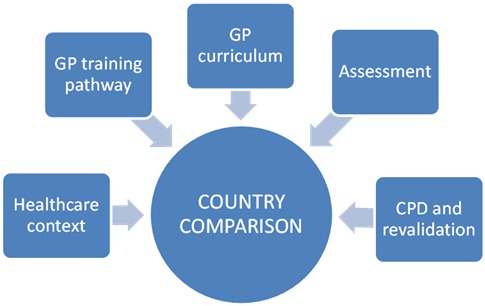
Overview of the key domains covered within the mapping framework

Findings arising from review of the RCGP draft mapping questions, from document scoping and from stakeholder interviews, informed the domains and questions included in a preliminary data collection template. Five key domains were identified: healthcare context, GP training pathway, GP training curriculum, GP training assessment, CPD and revalidation (Figure 3).

There was agreement between the research team and the RCGP that each of these domains should be included in the mapping process. Further discussion identified specifically how the curriculum and how requirements for remaining up-to-date should be mapped.

Discussion centred around which curriculum version GPs had qualified under and how to accommodate different cohorts of qualifying GPs. It was agreed that the most recent GP curriculum in the comparison country would be mapped to the most recent version of the UK GP curriculum from 2016,^11^ and that the UK’s current CPD and revalidation requirements would be used.^12^ It was acknowledged that CEGPR applicants were likely to have qualified under older curricula, but the rationale for selecting only the most recent curriculum included: a) that it was not feasible within the time-frame of this work to map past curricula or other requirements in addition to the current ones, and b) local CPD and revalidation (or relicensing) requirements should ensure that a GP is up-to-date and fit to practise. As such, the use of the current curriculum in conjunction with CPD and revalidation requirements was deemed to provide an indication of a GP’s current knowledge and skills (at time of CEGPR application), rather than those gained on initial completion of their GP training. Mapping the current standards and requirements to practise would therefore give an indication of whether streamlining of the CEGPR process was suitable for each country mapped.

A further issue relating to the curriculum domain was the level of detail that the mapping would include. The RCGP curriculum (2016) consists of 1) core statement: being a general practitioner,^11^ 2) professional modules,^10^ and 3) clinical modules.^10^ Stakeholder interviewees emphasised the importance of mapping the breadth of the curriculum, and the necessity of focusing on the core statement.

**Figure 4:**
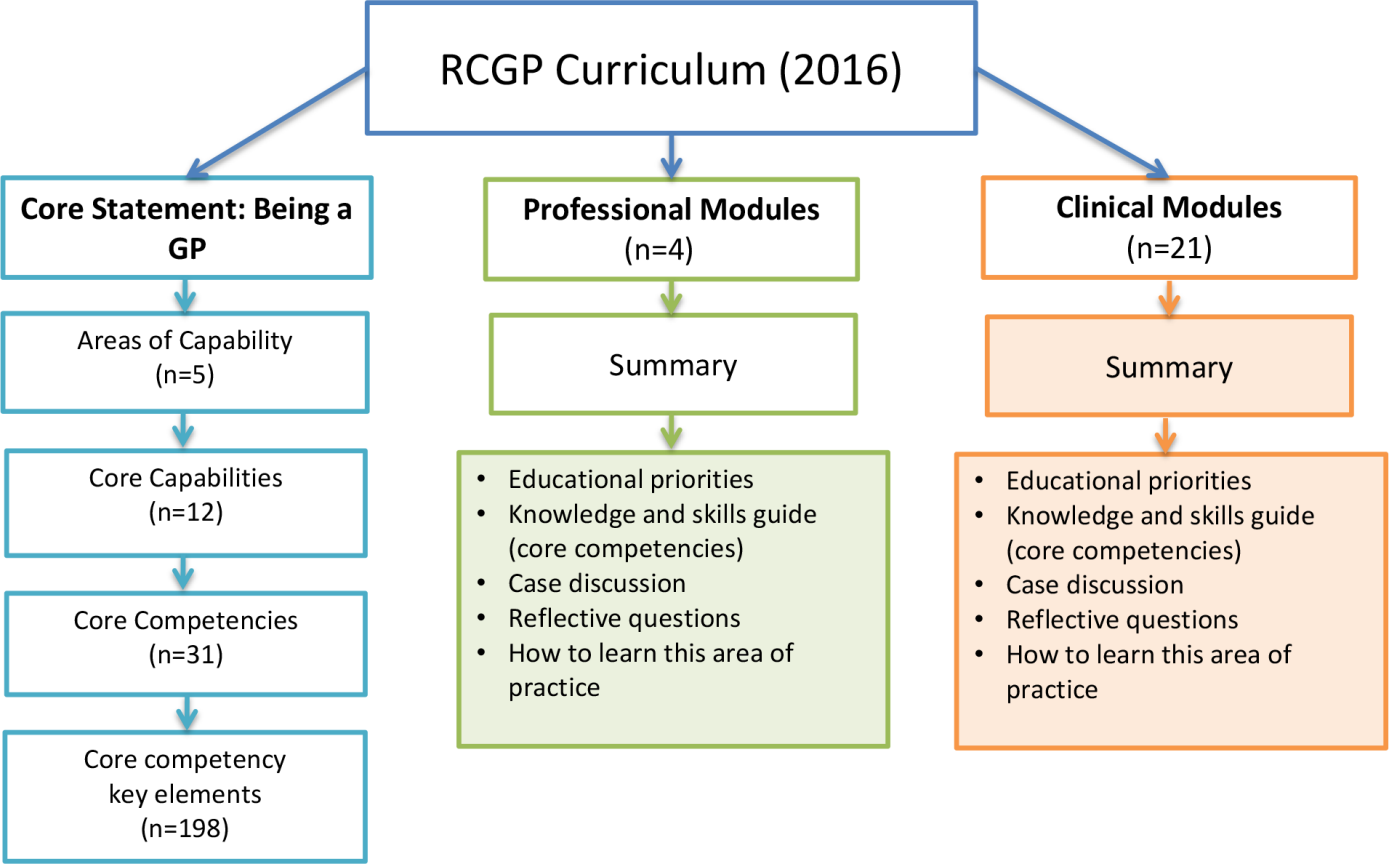
Structure and overview of content of the RCGP 2016 curriculum *n* represents the number of items within each section of the curriculum. Mapping was undertaken at the level of the unshaded boxes only.

Figure 4 illustrates the level of detail with which the curriculum mapping was undertaken. The core statement was mapped to the level of all 198 elements of the core competencies (further information available from the author on request). Professional modules were mapped to their summary descriptions. Clinical modules were mapped in terms of inclusion or exclusion of the clinical topic, but the content was not mapped in detail. The rationale for this last decision was that the curriculum notes that the provided list of clinical topics represents examples of what *might* be included in the GP role, but that the list (while comprehensive) is not explicitly intended to be exhaustive.

### Stage 2: Case study

The detailed results of the case study are beyond the scope of this paper. However, the process of completing the case study enabled the researchers to pilot and refine the methodology, including the application of the alignment scores and RAG rating processes. For example, mapping questions that were very similar or related were combined, and alignment scores and RAG ratings were applied at the level of each individual mapping question to help with applying scores and RAG ratings at the level of overarching domains. The alignment rating provided a RAG rating for each of the domains and for the country overall. The results for Australia were green RAG ratings for the domains of: GP training pathway, curriculum, and assessment; and amber RAG ratings for the domains of healthcare context, and CPD and revalidation. Australia had an overall RAG rating of green, suggesting it was suitable for consideration of a streamlined CEGPR process.

### Stage 3: Refining the data collection template

Review and refinement of the mapping questions produced a refined data collection template with clear question parameters, appropriate sections and subsections, and no extraneous questions. All of the five original domains were retained and a total of 49 mapping questions were generated across the domains (further information available from the author on request).

### Stage 4: Creating the mapping framework

**Figure 5:**
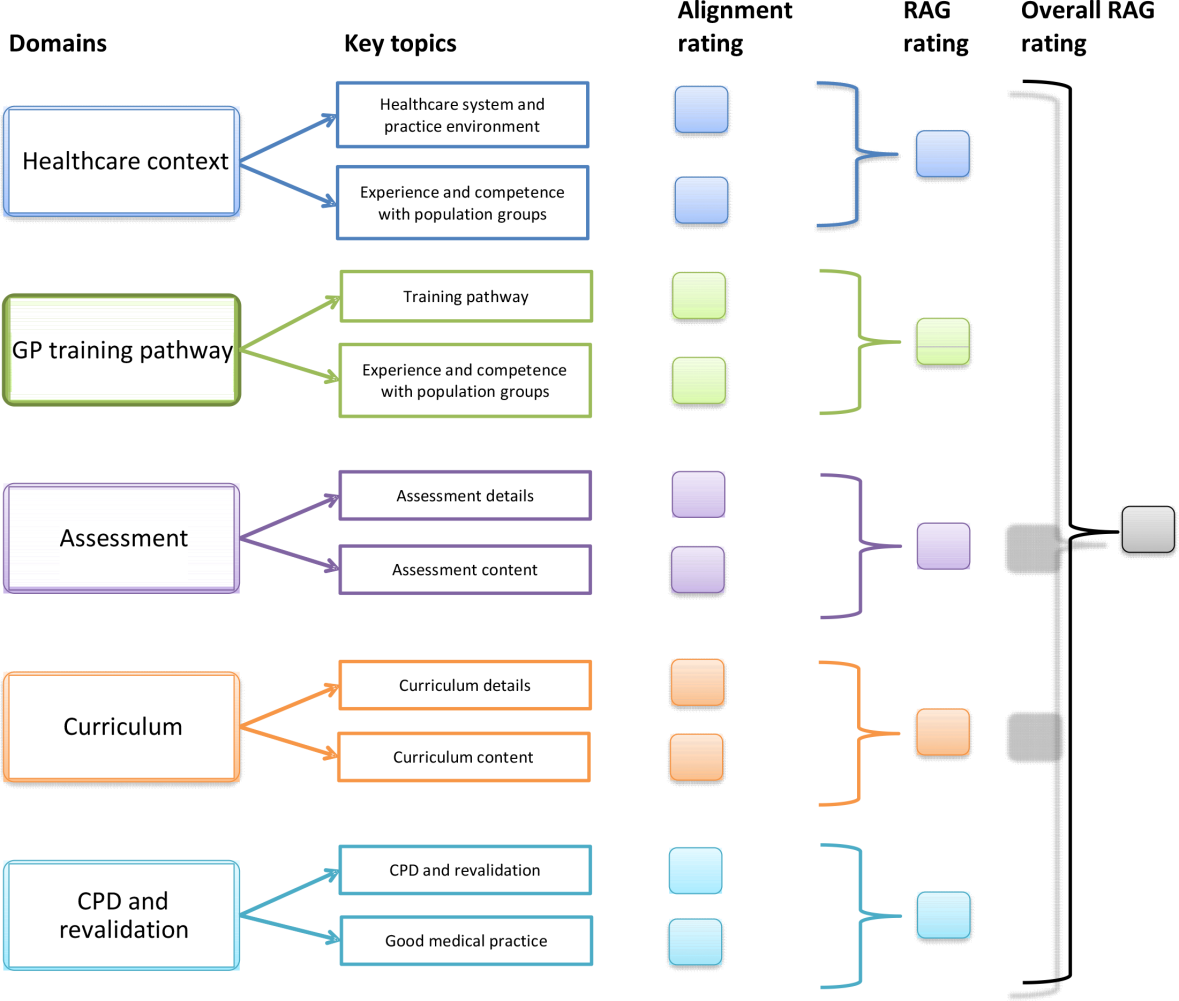
Structure and content of the proposed mapping framework (covering all five key domains) Colour coding indicates which key topics are contained within each of the five mapping domains

The refined template, the mapping questions, and the RAG rating system were used to generate the proposed final mapping framework (Figure 5). The framework comprises the mapping domains, alignment ratings for the key (combined) sections within these, RAG ratings for each domain, and an overall country RAG rating. The content of the domains is available from the author on request.

In addition, two further alternative mapping frameworks, both based on the one presented in this publication, were created for potential use in any future mapping work: 1) a simplified framework, and 2) a framework that included mapping of each domain in more detail. The three frameworks together offer potential flexibility of the mapping methodology.

## Discussion

### Summary

At a time of increased, targeted international recruitment of GPs, any changes to equivalence routes to the UK’s GP register must be informed by robust methods of comparison between different countries and the UK. The authors developed and reported a screening tool to help inform specifically whether a country could be considered for a streamlined CEGPR process, using a comprehensive yet easily interpreted and illustrated RAG rating system. A rapid review was undertaken of existing policies, procedures, and publications relevant to informing the design of a practical approach to assessing the comparability of GP education and training in another country with the UK. A test case was used to refine the tool and to assess its plausibility.

The final version of the tool provides an indication of issues and topics that might require more or less evidence and exploration within a streamlined CEGPR decision for an individual country. This, in conjunction with the known generic reasons why some candidates are unsuccessful in their CEGPR application,^9^ provides evidenced foundations for the formation of streamlined CEGPR requirements. The authors believe that the methodology described is scalable, and that the comprehensive and detailed exploration of country domains is fair. Fairness and reliability could be further explored through an assessment of inter-rater reliability of the alignment and RAG ratings scoring, and the use of a country expert to review and comment on a country’s case study results.

### Strengths and limitations

The main strength of this work was the use of an experienced academic team who were able to ground the project in clinical, primary care, educational, international, and methodological expertise. This work offers a robust methodology to support the agenda of international recruitment of GPs. The team achieved rapid turnaround of the work, aided by effective communication and collaborative working with the RCGP. The team also considered whether the mapping framework could be applied to other countries and has identified, in advance, issues that might arise when, for example, mapping a country where comparable healthcare context statistics are difficult to find, or where there may be sensitivity in approaching expert key informants. A potential limitation of this work was the necessity to focus on England, rather than reporting separately on each of the UK’s four nations, where different health systems have evolved since devolution (although professional regulation and education remain UK-wide).

### Comparison with existing literature

Published work exists relating to comparison of primary school education curricula in England to those of other countries,^13^ and also relating to comparison of healthcare workforce metrics from international perspective,^14^ but to the authors’ knowledge, no prior work has explored the potential for mapping GP training between different countries and the UK.

### Implications for research and practice

Reporting the complexity of UK healthcare context had to be carefully managed through use of clear questions and informed decisions about the scope and requirements of the work. In addition, although both countries involved in this work had English as their first language, care had to be taken to avoid any misinterpretation of language and concepts across cultures. Consequently, it is recommended that any future application of this work also includes a review of each country’s mapping report by a country expert. An additional challenge for any further application of this work is accommodating potential changes within the domains being explored. For example, current changes to CPD requirements (Australia), and known future changes to curricula (UK) illustrate the need for detailed and current country-level understanding.

The aim of a streamlined CEGPR process for GPs from non-EEA countries would be to make the application process quicker and less burdensome for applicants and assessors, without compromising patient safety and GP registration standards. Enabling timely recruitment of overseas GPs could, in turn, help to ease the overall system-wide pressures currently being experienced in UK general practice. An increase in doctors coming into the UK from overseas could also provide greater opportunities in the future for senior UK doctors to deliver training and mentoring support, thus expanding their role and contribution to the workforce. This work provides reassurance that a systematic methodology for mapping GP training between different countries and the UK has been developed, with the potential for these methods to be applied to other specialties looking to recruit internationally. The methodology developed may also be of use in the context of recent work by WONCA (World Organization of Family Doctors) Europe and its teaching organisation, EURACT, to define the general practice/family medicine specialty within the EU region.^14^ In addition, at a time of changing workforce dynamics globally, it will be important to open up these practical issues to academic rigour.

Any changes to the current CEGPR and overseas GP recruitment processes will have implications for the key statutory, regulatory, and professional agencies responsible for medical training and registration and, as such, these agencies would need to have sight and input into any future policies in this area.
